# Fueling the Fire: How Glutamine Metabolism Sustains Leukemia Growth and Resistance

**DOI:** 10.3390/biomed6010007

**Published:** 2026-02-04

**Authors:** Giovannino Silvestri

**Affiliations:** 1 Marlene and Stewart Greenebaum Comprehensive Cancer Center, Baltimore, MD 21201, USA; 2 Member Cancer Therapeutics Program, Marlene and Stewart Greenebaum Comprehensive Cancer Center, Baltimore, MD 21201, USA; 3 Department of Medicine, University of Maryland School of Medicine, Baltimore, MD 21201, USA

**Keywords:** glutamine metabolism, leukemia, acute myeloid leukemia (AML), glutaminase inhibition, glutamine addiction, cancer metabolism, IGF2BP2, SLC1A5, leukemic stem cells, redox balance, therapeutic resistance, metabolic targeting, FLT3-ITD

## Abstract

Glutamine metabolism has emerged as one of the most critical bioenergetic and biosynthetic programs sustaining leukemic cell growth, survival, stemness and therapeutic resistance. In both acute and chronic leukemias, including acute myeloid leukemia (AML) and acute lymphoblastic leukemia (ALL), malignant cells display a strong dependency on extracellular glutamine to support mitochondrial respiration, anabolic biosynthesis and redox homeostasis. This dependency is reinforced by oncogenic signaling networks, post-transcriptional metabolic regulation and microenvironmental adaptation within the bone marrow niche. Therapeutic strategies targeting glutamine utilization, including glutaminase inhibition, transporter blockade and enzymatic glutamine depletion, have demonstrated robust antileukemic activity in preclinical models, and early clinical efforts have begun to explore glutamine-directed interventions in myeloid neoplasms. However, metabolic plasticity, microenvironment-derived nutrient buffering and systemic toxicity remain significant limitations to clinical translation. This review provides a detailed synthesis of the biochemical framework of glutamine metabolism in leukemia, the molecular mechanisms enforcing glutamine addiction, the downstream functional consequences on proliferation, redox balance and leukemic stem cell biology, the current landscape of therapeutic strategies and emerging directions aimed at overcoming resistance and improving clinical efficacy.

## 1. Introduction

Metabolic reprogramming is now firmly established as a core hallmark of malignant transformation. While the historical focus on aerobic glycolysis—originally described by Warburg—remains foundational, it has become increasingly evident that cancer cells rely on a broader metabolic network that extends well beyond glucose utilization. Among non-glucose nutrients, glutamine has emerged as a particularly critical substrate, serving simultaneously as a carbon source for mitochondrial metabolism, a nitrogen donor for biosynthesis, and a regulator of cellular redox balance [1,2]. This multifaceted role positions glutamine as a central metabolic hub in cancer biology.

Leukemias represent a particularly compelling context in which to study glutamine metabolism. These malignancies arise within the bone marrow, a metabolically complex and spatially heterogeneous environment characterized by fluctuating oxygen tension, variable nutrient availability, and intense competition between malignant and normal hematopoietic cells [3,4]. Leukemic cells must proliferate rapidly, survive genomic and oxidative stress, and endure sustained exposure to cytotoxic chemotherapy, targeted inhibitors, and immune-based therapies. These demands impose a high metabolic burden that cannot be met by glycolysis alone.

A substantial body of metabolomic and functional evidence indicates that many, but not all, leukemias exhibit functional dependence on glutamine. This dependence varies by genetic subtype, differentiation state, treatment history, and microenvironmental context, and is therefore best understood as a conditional rather than universal feature of leukemic metabolism.

Early observations demonstrated that plasma glutamine levels are significantly reduced in patients with acute leukemia, reflecting excessive consumption by malignant cells [5–7]. Subsequent studies showed that leukemic blasts and leukemic stem cells (LSCs) exhibit enhanced glutamine uptake, elevated expression of glutamine transporters, and increased flux through glutaminolytic pathways [8–10]. Importantly, disruption of glutamine metabolism consistently induces apoptosis in acute myeloid leukemia (AML) and acute lymphoblastic leukemia (ALL) models, establishing glutamine addiction as a bona fide metabolic vulnerability. Importantly, glutamine dependency is not uniform across leukemias or even across AML cases. The magnitude and functional importance of glutamine utilization vary with genetic context, differentiation state, treatment history, and microenvironmental conditions. Accordingly, in this review “glutamine addiction” is treated as a context-restricted vulnerability rather than a universal property of leukemic cells.

Functionally, glutamine can be indispensable in leukemias that are metabolically constrained—e.g., subsets with high mitochondrial/OXPHOS reliance, impaired compensatory nutrient pathways, or enforced extracellular dependence—where glutamine withdrawal or glutaminase blockade triggers energetic collapse and oxidative stress [8,11–13]. By contrast, in more metabolically plastic settings (including therapy-adapted states), glutamine can be replaceable, with cells maintaining growth and survival through increased fatty acid oxidation, one-carbon metabolism, or alternative amino acid fueling [11–13]. Thus, glutamine dependence is best framed as a state that emerges from genetic, niche, and treatment context rather than a uniform requirement across leukemias [11–13].

Beyond its metabolic utility, glutamine plays critical roles in shaping therapy response. Mitochondrial glutamine metabolism supports oxidative phosphorylation (OXPHOS), which has been linked to chemoresistance, persistence of minimal residual disease, and survival of quiescent LSCs [11–13]. Additionally, glutamine-derived glutathione acts as a dominant antioxidant buffer, protecting leukemic cells from reactive oxygen species (ROS) generated by both mitochondrial metabolism and anticancer drugs [8]. These functions directly connect glutamine metabolism to disease relapse and treatment failure.

At the same time, glutamine is indispensable for non-malignant cells, including cycling lymphocytes, intestinal epithelium, and stromal populations within the marrow niche [14–16]. Immune cells, in particular, utilize glutamine to fuel proliferation and effector function, creating a therapeutic paradox: systemic glutamine depletion may impair anticancer immunity even as it suppresses leukemia growth [15,17]. This duality necessitates a nuanced understanding of how glutamine metabolism is differentially regulated and exploited in leukemic versus normal cellular compartments.

The aim of this review is therefore threefold. First, we provide a detailed overview of the biochemical pathways governing glutamine metabolism in leukemia. Second, we examine molecular mechanisms—oncogenic, epigenetic, and microenvironmental—that actively enforce glutamine addiction. Third, we synthesize preclinical and clinical efforts to therapeutically target glutamine metabolism, highlighting limitations, resistance mechanisms, and future directions. By integrating metabolic biology with leukemia pathogenesis, this review seeks to clarify how glutamine fuels disease progression and how this dependency might be exploited for durable therapeutic benefit.

## 2. Glutamine Biochemistry and Its Central Importance in Leukemic Cells

### 2.1. Cellular Uptake and Compartmentalization of Glutamine

Glutamine is the most abundant free amino acid in human plasma, maintained at concentrations of approximately 0.6–0.8 mM under physiological conditions, where it functions as a systemic nitrogen carrier linking muscle, liver, lung, and immune tissues [1,18]. Rapidly proliferating leukemic cells exploit this circulating reservoir to satisfy increased anabolic, bioenergetic, and redox demands imposed by malignant growth and therapeutic stress.

In leukemia, glutamine uptake is primarily mediated by the sodium-dependent neutral amino acid transporter SLC1A5, which functions as the dominant conduit for extracellular glutamine influx. Elevated SLC1A5 expression has been documented in both AML and ALL patient samples and is associated with aggressive disease features and poor clinical outcomes [10,19,20]. Genetic ablation or pharmacologic inhibition of SLC1A5 markedly reduces intracellular glutamine and glutamate pools, leading to mitochondrial dysfunction, oxidative stress, and apoptotic cell death [10,20,21]. These findings establish SLC1A5 as a central gatekeeper of glutamine entry in leukemic cells.

However, glutamine transport in leukemia is not mediated by a single transporter. Additional systems, particularly members of the SLC38 (SNAT) family, contribute to maintaining intracellular glutamine homeostasis, especially under conditions of fluctuating nutrient availability within the bone marrow niche [22]. These transporters can partially compensate when SLC1A5 function is limited, thereby conferring metabolic flexibility and buffering leukemic cells against acute glutamine deprivation.

A critical functional interrelationship exists between glutamine influx and anabolic signaling through the LAT1 (SLC7A5)–SLC3A2 heterodimeric transporter complex. LAT1 operates as an obligate amino acid exchanger, importing essential amino acids such as leucine in exchange for intracellular glutamine. This exchange mechanism couples glutamine availability to activation of mTORC1, thereby reinforcing protein synthesis, mitochondrial biogenesis, and proliferative growth programs [23,24]. Consequently, intracellular glutamine not only serves as a metabolic substrate but also functions as a signaling currency that links nutrient uptake to oncogenic growth pathways.

Following cytosolic entry, glutamine is partitioned between protein synthesis and catabolic metabolism. A substantial fraction is transported into mitochondria via specialized carriers that are only beginning to be molecularly defined [25]. Within the mitochondrial matrix, glutamine becomes a key substrate for energy production, redox control, and biosynthetic precursor generation, thereby supporting multiple metabolic processes critical to leukemic cell survival.

The major glutamine transporters involved in leukemic glutamine uptake, their directionality, and their functional coupling relationships are summarized in Table 1 [10,22–24].

The biochemical steps outlined in this section establish the metabolic framework upon which the functional consequences described in Section 4 are built.

### 2.2. Anaplerosis, Reductive Carboxylation and Biosynthesis

The canonical fate of mitochondrial glutamine is conversion to glutamate by glutaminase (GLS), followed by transformation into α-ketoglutarate (α-KG) through glutamate dehydrogenase (GLDH) or aminotransferases [1,18,26]. α-KG enters the tricarboxylic acid (TCA) cycle, replenishing carbon intermediates lost to biosynthetic reactions—a process known as anaplerosis. This function is particularly crucial in leukemia, where citrate is siphoned for lipid synthesis and oxaloacetate and aspartate are diverted toward nucleotide biosynthesis [26,27].

Glutamine also provides the amide nitrogen required for de novo synthesis of purines and pyrimidines, feeding into phosphoribosyl pyrophosphate and carbamoyl phosphate-dependent reactions [26,28]. As a result, glutamine deprivation rapidly impairs DNA and RNA synthesis, selectively targeting proliferative leukemic cells while sparing quiescent normal counterparts.

Under hypoxic conditions—prevalent within the bone marrow niche—leukemic cells can further adapt glutamine metabolism through reductive carboxylation. In this pathway, α-KG is converted back into citrate via reverse isocitrate dehydrogenase activity, sustaining lipid biosynthesis independently of glucose oxidation [29,30]. This metabolic plasticity allows leukemic cells to maintain anabolic output even when oxygen availability restricts classical oxidative phosphorylation.

### 2.3. Redox Regulation and Glutathione Synthesis

Beyond carbon and nitrogen metabolism, glutamine plays a central role in regulating intracellular redox balance. Glutamate derived from glutamine is a rate-limiting precursor for synthesis of glutathione (GSH), the dominant intracellular antioxidant [8,31]. GSH detoxifies ROS generated by mitochondrial respiration, oncogenic signaling, and anticancer therapies, thereby preventing oxidative damage to DNA, proteins, and lipids.

In AML models, inhibition of glutaminase leads to depletion of intracellular GSH, accumulation of mitochondrial ROS, and activation of intrinsic apoptotic pathways [25,32,33]. These effects convert glutamine metabolism from a survival mechanism into a therapeutic vulnerability, particularly when combined with agents that further elevate ROS levels. Collectively, the biochemical functions outlined above explain why glutamine metabolism occupies a uniquely central position in leukemic cell biology. A consolidated overview of glutamine uptake and its major metabolic fates in leukemia cells is provided in Table 2 [1,18,26,28–31].

Collectively, the pathways summarized above form the biochemical basis for the multiple biological roles of glutamine in leukemia, which are consolidated in Table 3.

## 3. Mechanisms Enforcing Glutamine Dependency in Leukemia

Glutamine addiction in leukemia is not merely a passive reflection of increased proliferation. Instead, it is actively imposed and maintained through oncogenic signaling networks, post-transcriptional regulators, and metabolic adaptations to therapeutic stress and microenvironmental constraints.

### 3.1. Transcriptional Control by Oncogenes and Stress Pathways

The MYC proto-oncogene is a master regulator of glutamine metabolism across cancer types. MYC directly induces transcription of SLC1A5, GLS1, and multiple enzymes involved in nucleotide and serine biosynthesis, thereby coordinating glutamine uptake with anabolic demand [32–34]. In AML, MYC activation—whether through chromosomal abnormalities or upstream signaling—correlates with elevated glutamine flux and heightened sensitivity to glutaminase inhibition [34].

mTORC1 signaling constitutes a second critical axis linking glutamine to cell growth. Glutamine promotes mTORC1 activation indirectly by facilitating LAT1-mediated leucine uptake and directly through Rag GTPase–dependent signaling [23,24]. mTORC1 enhances translation of metabolic enzymes and mitochondrial proteins, creating a feed-forward loop that amplifies glutamine utilization and mitochondrial activity.

Leukemia cells must also adapt to environmental stress. Hypoxia stabilizes HIF-1α and HIF-2α, which reprogram metabolism toward glycolysis but also modulate glutamine metabolism by influencing transaminase expression and α-KG utilization [30]. Concurrently, activation of the integrated stress response through ATF4 promotes expression of amino acid transporters and enzymes, enabling cells to weather nutrient scarcity [37]. Together, these pathways ensure sustained glutamine availability under fluctuating marrow conditions.

### 3.2. Post-Transcriptional and Epigenetic Regulation: The IGF2BP2 Axis

A major conceptual advance in leukemia metabolism emerged with the identification of insulin-like growth factor 2 mRNA-binding protein 2 (IGF2BP2) as a post-transcriptional regulator of glutamine metabolism. Weng and colleagues demonstrated that IGF2BP2 functions as an epigenetic modifier m^6^A reader that stabilizes transcripts encoding SLC1A5, MYC, and glutamine-processing enzymes [38]. Loss of IGF2BP2 reduces glutamine uptake and flux, impairs mitochondrial metabolism, and selectively diminishes leukemic stem cell self-renewal in serial transplantation models.

Crucially, IGF2BP2 deletion has limited impact on normal hematopoietic stem cells, suggesting a therapeutic window. High IGF2BP2 expression correlates with poor prognosis in AML, positioning this regulator as both a metabolic driver and a potential biomarker [38]. Pharmacologic disruption of IGF2BP2-mediated RNA binding further validates this pathway as an upstream target capable of simultaneously suppressing multiple glutamine-dependent processes.

### 3.3. Enforced Glutamine Dependency via Loss of Synthesis Pathways

Some leukemias further reinforce glutamine addiction by suppressing endogenous glutamine synthesis. In NOTCH1-driven T-ALL, glutamine synthetase (GS) expression is markedly reduced, eliminating the ability to generate glutamine from glutamate and ammonia [41]. These cells become obligately dependent on extracellular glutamine, rendering them exquisitely sensitive to glutamine depletion or ASCT2 inhibition. This enforced dependency illustrates how oncogenic signaling can hard-wire metabolic vulnerabilities into leukemia cells.

### 3.4. Therapeutic Stress and Adaptive Remodeling of Glutamine Metabolism

Targeted therapies frequently shift the metabolic landscape of leukemia. In FLT3-mutant AML, treatment with tyrosine kinase inhibitors such as midostaurin, gilteritinib or quizartinib reduces glycolytic flux and proliferation but can simultaneously enhance reliance on mitochondrial respiration. Preclinical work suggests that residual leukemic cells surviving FLT3 inhibition show increased glutaminase expression, enhanced glutamine oxidation and higher dependence on OXPHOS for survival, effectively using glutamine metabolism as an adaptive resistance mechanism [42].

Khamari and colleagues recently demonstrated that persistent AML cells under targeted therapy maintain metabolic flexibility through glutamine metabolism, and that pharmacologic inhibition of GLS1 sensitizes these cells to kinase inhibitors both in vitro and in mouse models [43]. These studies argue strongly that glutamine metabolism is not only a baseline vulnerability but also a dynamic mediator of therapy resistance.

**Figure 1.**
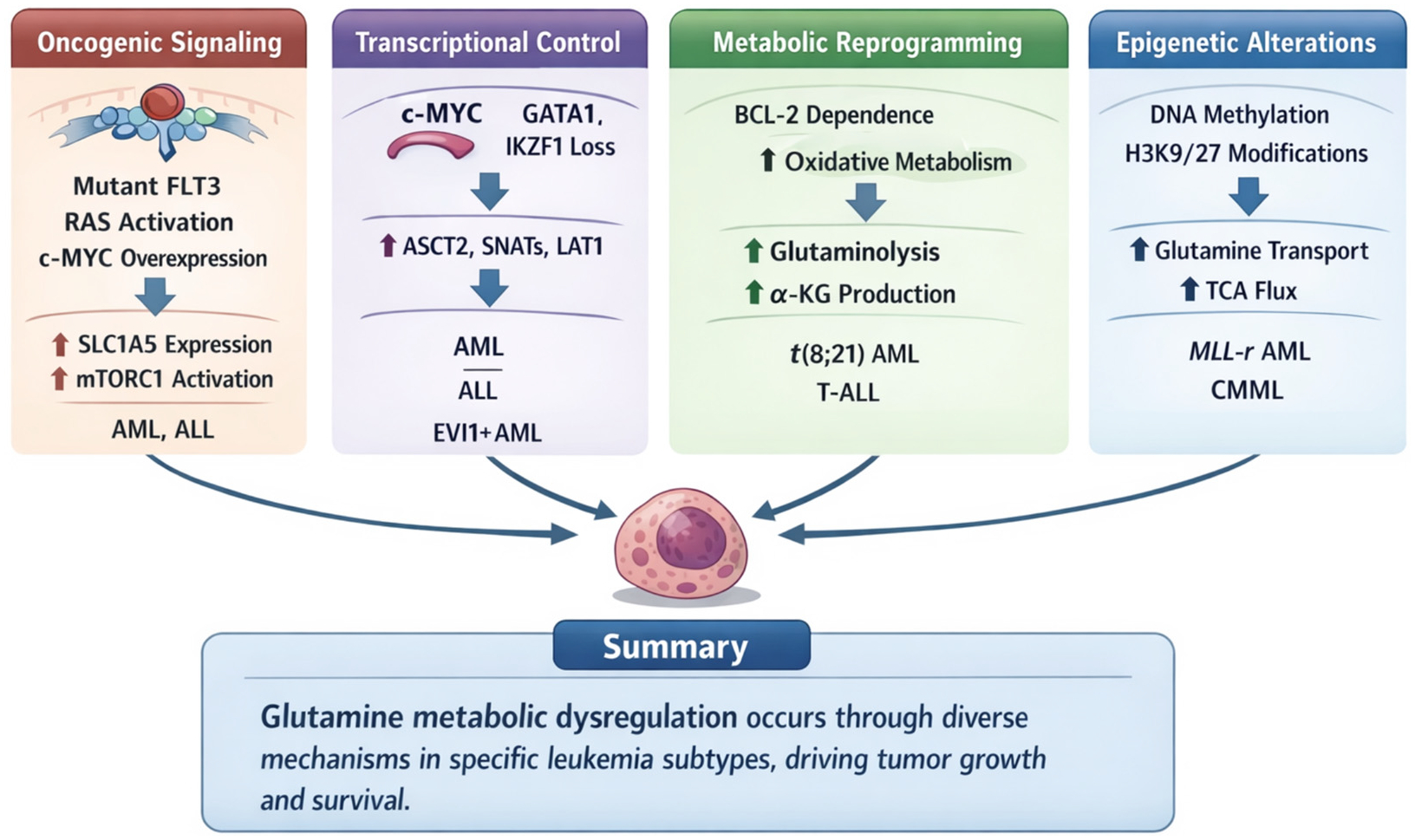
Dysregulation of Glutamine Metabolism in Leukemia.

Glutamine dependency in leukemia is actively imposed by oncogenic transcription, post-transcriptional RNA regulation, suppression of synthesis pathways, and therapy-induced metabolic rewiring. These regulatory layers operate in subtype- and state-specific patterns rather than uniformly across leukemias, as summarized in Figure 1 [23,24,34,37,38,41–44].

Collectively, glutamine dependence in leukemia is enforced by (i) oncogenic demand programs (e.g., MYC/mTORC1), (ii) post-transcriptional stabilization (IGF2BP2), (iii) loss of synthetic capacity (GS suppression in NOTCH1-driven T-ALL), and (iv) therapy-induced metabolic rewiring (e.g., FLT3 inhibitor–treated residual AML). Importantly, these mechanisms also define predictable failure boundaries, because leukemias with high metabolic plasticity can bypass glutamine restriction via FAO and related compensatory pathways [11–13,23,24,34,37,38,41–45].

## 4. Functional Consequences of Glutamine Metabolism in Leukemia

Glutamine metabolism supports multiple functional programs in leukemic cells, including mitochondrial bioenergetics, redox control, epigenetic regulation, and maintenance of stem-like properties; however, the relative contribution of these processes varies substantially across leukemia subtypes, disease stages, and treatment contexts. While several studies demonstrate that glutamine-derived carbon and nitrogen flux sustain proliferation and survival in subsets of acute myeloid leukemia (AML) and acute lymphoblastic leukemia (ALL), emerging evidence indicates that glutamine dependency is neither universal nor static and can be bypassed through metabolic rewiring under therapeutic or microenvironmental pressure [11–13]. Accordingly, Section 4 focuses on downstream functional outputs of these pathways—including bioenergetics, redox regulation, epigenetic state, and stem-like persistence—rather than re-describing the biochemical steps detailed in Section 2 [11–13,26,31,35,36,46].

### 4.1. Proliferation, Bioenergetics and Cell Cycle Progression

One of the most direct consequences of glutamine metabolism in leukemia is its role in sustaining cellular bioenergetics. Through conversion to α-ketoglutarate, glutamine fuels the tricarboxylic acid (TCA) cycle and supports OXPHOS, which is increasingly recognized as a dominant energy source in AML cells [11–13]. In contrast to normal hematopoietic progenitors, which retain metabolic flexibility, leukemic blasts often exhibit a restricted reliance on mitochondrial metabolism, rendering them vulnerable to glutamine deprivation.

Experimental inhibition of glutaminase rapidly depletes intracellular α-KG pools, leading to reduced oxygen consumption, ATP depletion, and suppression of cell-cycle progression [34,45]. In AML cells, these effects manifest as G1 arrest followed by apoptotic cell death. Importantly, glutamine restriction preferentially affects leukemic cells with high mitochondrial mass and respiration, a phenotype frequently associated with poor prognosis and chemotherapy resistance [42,43].

In genetically defined subtypes, glutamine metabolism becomes even more critical. IDH1- and IDH2-mutant AML cells are uniquely dependent on glutamine-derived α-KG as substrate for neomorphic IDH enzymatic activity that generates the oncometabolite 2-hydroxyglutarate (2-HG) [34–36]. Inhibition of glutaminase reduces 2-HG levels, disrupts aberrant epigenetic programming, and promotes partial differentiation of leukemic blasts [34]. These findings directly link glutamine metabolism to both oncogene-driven bioenergetics and differentiation blockade.

Notably, reliance on glutamine-fueled oxidative phosphorylation is not conserved across all leukemias. Drug-resistant ALL and metabolically adapted AML populations have been shown to reduce glutamine utilization and compensate through enhanced fatty acid oxidation, branched-chain amino acid catabolism, or increased glycolytic flux [11–13,46]. These findings indicate that glutamine-driven bioenergetics reflects a constrained metabolic state rather than an obligate requirement, reinforcing the need to define when and where glutamine metabolism constitutes a functional vulnerability.

### 4.2. Redox Homeostasis and DNA Damage Responses

A second major functional outcome of glutamine metabolism is the maintenance of redox equilibrium. Leukemic cells generate high levels of reactive oxygen species (ROS) as a consequence of active mitochondrial respiration, oncogenic signaling, and oxidative DNA replication stress [47,48]. Glutamine-derived glutamate is essential for synthesis of GSH, the primary intracellular antioxidant that buffers ROS and prevents oxidative damage.

Multiple studies demonstrate that inhibition of glutamine metabolism leads to depletion of GSH, accumulation of mitochondrial ROS, and activation of intrinsic apoptotic pathways in AML cells [8,32,33]. These effects extend to leukemic stem cells, which rely heavily on antioxidant defenses to survive chemotherapy-induced oxidative stress [42,43]. Disruption of glutathione synthesis therefore converts a protective metabolic adaptation into a therapeutic vulnerability.

Importantly, glutamine metabolism intersects with broader cellular antioxidant systems, including glutathione peroxidase (GPX), thioredoxin, and peroxiredoxin networks [48]. In ALL, redox imbalance contributes directly to leukemogenesis, and suppression of ROS-generating enzymes has been proposed as a complementary therapeutic approach [47]. The tight integration between glutamine metabolism and redox control explains why glutaminase inhibition synergizes with ROS-inducing agents and why metabolic targeting can overcome otherwise refractory disease states.

However, leukemic cells may partially circumvent glutathione depletion by activating alternative antioxidant systems, including thioredoxin- and peroxiredoxin-dependent pathways, or by attenuating mitochondrial activity, thereby limiting the durability of glutamine-targeted redox strategies [32,33,47,48].

### 4.3. Epigenetic Regulation and Leukemic Differentiation

Glutamine metabolism also influences leukemia through epigenetic regulation. α-KG produced from glutamine is a required cofactor for α-KG–dependent dioxygenases, including TET family DNA demethylases and Jumonji-C domain histone demethylases [35]. These enzymes govern chromatin accessibility, lineage commitment, and transcriptional programs critical for hematopoietic differentiation.

In leukemias characterized by impaired differentiation, alterations in glutamine metabolism can either exacerbate or relieve epigenetic repression. In IDH-mutant AML, excess glutamine-derived α-KG is converted to 2-HG, which competitively inhibits dioxygenases and enforces a hypermethylated, differentiation-blocked state [36]. Targeting glutamine metabolism reduces 2-HG levels and partially restores normal epigenetic dynamics [34].

More broadly, metabolic restriction of glutamine alters histone and DNA methylation profiles even in IDH–wild-type AML, suggesting that direct manipulation of nutrient flux can remodel the epigenome independently of genetic mutations [38]. This connection provides a mechanistic explanation for the observed differentiation effects of metabolic therapies and highlights glutamine metabolism as an upstream regulator of leukemia cell fate.

Outside genetically defined contexts such as IDH-mutant AML, the epigenetic consequences of glutamine restriction are more variable and modest, suggesting that metabolic modulation alone is unlikely to uniformly restore differentiation programs across leukemias [34–36,38].

### 4.4. Leukemic Stem Cells, Minimal Residual Disease and Relapse

Leukemic stem cells (LSCs) represent the cellular reservoir responsible for disease persistence and relapse. Unlike bulk leukemic blasts, LSCs are relatively quiescent, exhibit high mitochondrial mass, and depend strongly on oxidative metabolism rather than glycolysis [39,40]. Leukemic stem cells represent heterogeneous populations defined by phenotype, functional self-renewal capacity, metabolic state, and genetic background. Consequently, reported glutamine dependency reflects diverse stem-like cellular states rather than a single conserved metabolic program. While some stem-enriched populations exhibit strong mitochondrial and glutamine dependence, others display substantial metabolic flexibility, maintaining survival through fatty acid oxidation, autophagy-mediated nutrient recycling, or stromal nutrient support within the bone marrow niche [11–13,38–40].

Compelling evidence links glutamine metabolism directly to LSC maintenance. Genetic or pharmacologic inhibition of IGF2BP2 disrupts glutamine metabolic flux, impairs mitochondrial respiration, and selectively reduces LSC self-renewal capacity while sparing normal hematopoietic stem cells [38]. Likewise, NOTCH1-driven T-ALL stem-like cells display enforced glutamine addiction due to suppression of glutamine synthetase, making them exquisitely sensitive to glutamine withdrawal [41].

**Figure 2.**
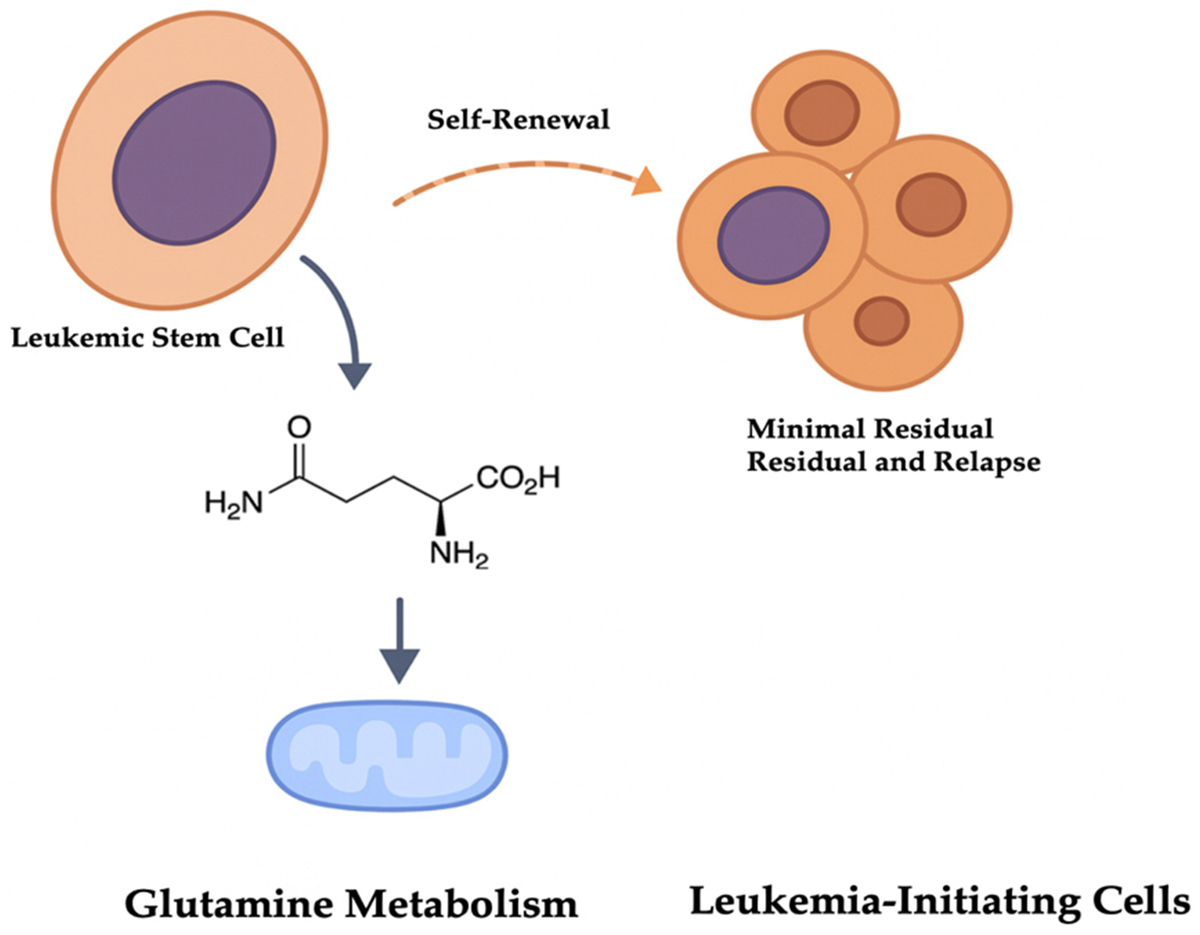
Glutamine metabolism sustains leukemic stem cell self-renewal and disease persistence. Leukemic stem cells rely on glutamine metabolism to support mitochondrial function and self-renewal. This metabolic dependency promotes the survival and expansion of leukemia-initiating cells, contributing to minimal residual disease and eventual relapse.

Collectively, available evidence supports glutamine metabolism as a vulnerability in selected stem-like leukemic states rather than a universal mechanism for eradication of minimal residual disease, underscoring the need for precise functional and genetic stratification in translational studies [4,38] (Figure 2).

Consistent with this heterogeneity, “LSC” definitions vary across studies (phenotypic gates vs. functional xenograft engraftment vs. MRD-associated stem-like transcriptional programs), which likely contributes to divergent conclusions regarding glutamine dependence and therapeutic selectivity [39,40]. Therefore, glutamine-targeting claims for MRD eradication should be interpreted in the context of the specific LSC assay and disease stage being modeled [39,40].

Because these functional outputs map directly onto therapeutic vulnerabilities (mitochondrial dependence, redox buffering, and stem-like persistence), the next section synthesizes current strategies to target glutamine metabolism and explains why resistance emerges in parallel [8,11–13,31,42,43,46].

## 5. Therapeutic Targeting of Glutamine Metabolism in Leukemia

Therapies aimed at disrupting glutamine metabolism have moved from conceptual proposals to concrete drug development, particularly within the class of glutaminase inhibitors. Table 4 summarizes key molecular regulators that represent potential therapeutic entry points, while Table 5 provides an overview of major strategies and representative agents.

### 5.1. Glutaminase Inhibition

Glutaminase inhibitors represent the most mature class of glutamine-targeted therapies. Telaglenastat (CB-839) selectively inhibits GLS1 and has demonstrated potent antileukemic activity in preclinical AML models [45]. CB-839 reduces glutamine-derived TCA flux, depletes GSH, increases oxidative stress, and induces apoptosis [8,25]. Gregory and co-workers extended these findings by showing that CB-839 treatment depletes intracellular GSH, elevates mitochondrial ROS and sensitizes AML cells to additional oxidative stress [8]. Subsequent studies in murine AML models confirmed that combining CB-839 with ROS-inducing agents such as arsenic trioxide or homoharringtonine produces strong synergistic antileukemic effects and reduces leukemic burden in vivo [56].

Clinical trials of CB-839 in hematologic malignancies are still in early phases but provide important insights. Konopleva and colleagues investigated telaglenastat in combination with azacitidine and venetoclax in relapsed or refractory myeloid malignancies. Although sample sizes remain small, the regimen was generally tolerable and showed encouraging hematologic responses [49]. A separate study reported that CB-839 improved metabolic and hematologic parameters in patients with myeloproliferative neoplasms, suggesting that glutaminase inhibition can be safely implemented in myeloid disease [50]. By contrast, a preclinical investigation in chronic lymphocytic leukemia found only limited single-agent activity of CB-839, underscoring disease-specific differences in glutamine dependence [51].

Despite encouraging early-phase tolerability and response signals, durable remissions and late-phase validation remain lacking. To date, no phase III trial has established glutaminase inhibition as a standard leukemia therapy, underscoring the gap between metabolic rationale and proven clinical benefit.

Newer glutaminase inhibitors, such as IPN60090, with improved pharmacokinetics and selectivity are entering clinical evaluation and may expand the therapeutic window for targeting glutamine metabolism [57].

### 5.2. Glutamine Depletion and Transporter Inhibition

Historically, L-asparaginase has been used in ALL therapy for its ability to deplete circulating asparagine, to which leukemic blasts are highly sensitive. Certain formulations of asparaginase also exhibit significant glutaminase activity and can reduce plasma glutamine levels. Clinical studies combining asparaginase with chemotherapy have shown substantial improvements in ALL outcomes, although it remains challenging to disentangle the relative contributions of asparagine versus glutamine depletion [53]. Nevertheless, these data provide proof of principle that systemic glutamine depletion is feasible and can produce therapeutic benefit, albeit at the expense of considerable hepatotoxicity, pancreatitis and immunogenic reactions.

SLC1A5 inhibitors such as V-9302 constitute a complementary strategy that acts at the level of glutamine uptake rather than systemic availability. Preclinical work in AML and ALL cell lines shows that V-9302 reduces glutamine import, diminishes TCA cycle flux and inhibits proliferation [52]. However, transport redundancy and upregulation of alternative carriers may limit the durability of this approach, making it more suitable for use in combination regimens rather than as monotherapy.

### 5.3. Rational Combinations with Targeted and Cytotoxic Agents

Growing evidence indicates that the most effective therapeutic applications of glutamine-targeted interventions will arise from rational drug combinations rather than single-agent approaches. Glutamine restriction imposes multifaceted metabolic stress on leukemic cells by impairing mitochondrial respiration, depleting antioxidant capacity, and limiting biosynthetic flux [10,31]. These vulnerabilities create opportunities to synergize glutamine-directed agents with therapies that converge on mitochondrial integrity, redox balance, and apoptotic priming.

One of the most compelling combination strategies pairs glutaminase inhibition with agents that enhance oxidative stress. By depleting glutathione and other glutamine-derived antioxidants, glutaminase inhibitors elevate intracellular reactive oxygen species (ROS), sensitizing leukemic cells to further ROS accumulation induced by drugs such as arsenic trioxide or homoharringtonine [8]. This redox-based synergy has been demonstrated in AML models, including leukemic stem cell–enriched populations, where combined treatment promotes mitochondrial damage, cytochrome c release, and intrinsic apoptosis more effectively than either agent alone [8,54]. Importantly, this strategy capitalizes on the heightened baseline oxidative stress and limited antioxidant reserve characteristic of leukemic cells compared with normal hematopoietic progenitors.

Combination with targeted kinase inhibitors represents another rational approach. In FLT3-mutant AML, treatment with FLT3 inhibitors suppresses proliferative signaling but frequently induces metabolic rewiring toward increased mitochondrial metabolism and glutamine utilization as an adaptive survival mechanism [54]. Glutaminase inhibition disrupts this metabolic escape route, reducing OXPHOS capacity and enhancing the depth and durability of responses to FLT3-directed therapy [54]. Similar principles apply to BCL-2 inhibition: venetoclax selectively targets mitochondria-dependent leukemic cells, and glutamine deprivation further compromises mitochondrial respiration and redox balance, lowering the apoptotic threshold [55]. These converging effects on mitochondrial fitness provide a mechanistic basis for combining glutaminase inhibitors with BCL-2 antagonists, particularly in AML subtypes characterized by high OXPHOS dependence.

Beyond direct cytotoxic synergies, glutamine-targeted strategies may enhance the efficacy of epigenetic and immune-based therapies. Metabolic restriction of glutamine alters α-ketoglutarate availability and redox status, which in turn can influence chromatin-modifying enzymes and transcriptional programs linked to differentiation and stemness [38,58]. Integrating glutamine metabolism inhibitors with epigenetic modulators may therefore reinforce leukemic differentiation or suppress self-renewal. Similarly, emerging evidence suggests that careful modulation of glutamine metabolism could be leveraged to improve immunotherapeutic outcomes, provided that sufficient metabolic support for effector immune cells is preserved. Achieving this balance will require precise dosing schedules, temporal sequencing, or cell-selective targeting strategies.

Collectively, these findings underscore that glutamine metabolism is best exploited therapeutically as part of an integrated strategy. Rational combinations that simultaneously constrain metabolic flexibility, amplify mitochondrial stress, and prevent adaptive rewiring are likely to yield the greatest clinical benefit.

## 6. Challenges, Limitations and Resistance Mechanisms

Despite robust preclinical evidence supporting glutamine metabolism as a therapeutic vulnerability in leukemia, several biological and translational challenges continue to limit the clinical efficacy of glutamine-targeted therapies. These challenges arise from intrinsic metabolic plasticity, extrinsic microenvironmental support, systemic toxicity, and inter- and intra-disease heterogeneity.

### 6.1. Metabolic Plasticity and Pathway Redundancy

One of the principal obstacles to durable glutamine-targeted therapy is the remarkable metabolic adaptability of leukemic cells. When glutamine availability or utilization is restricted, leukemia cells can activate compensatory pathways to maintain energy production and biosynthesis. Increased reliance on fatty acid oxidation has been documented as a resistance mechanism in several leukemia models, allowing sustained mitochondrial respiration in the face of amino acid limitation [11,12,37]. Similarly, enhanced flux through serine–glycine one-carbon metabolism or branched-chain amino acid catabolism can partially substitute for glutamine-derived carbon and nitrogen, supporting nucleotide synthesis and redox control [13,59–62].

Enzymatic redundancy further complicates glutamine targeting. Asparagine synthetase possesses intrinsic glutaminase activity and can contribute to intracellular glutamine catabolism independently of canonical GLS1 signaling [63]. Inhibition of GLS1 alone may therefore be insufficient to fully suppress glutamine-driven anaplerosis, particularly in leukemias with high ASNS expression. These compensatory and redundant pathways highlight the limitations of single-node metabolic inhibition and reinforce the rationale for combination strategies that concurrently suppress multiple nutrient inputs or downstream metabolic sinks.

### 6.2. Contexts Where Glutamine Targeting Fails or Loses Efficacy

Not all leukemias exhibit strong functional dependence on glutamine. Drug-resistant ALL models have demonstrated reduced reliance on exogenous glutamine, accompanied by metabolic rewiring toward alternative substrates such as fatty acids and serine–glycine one-carbon metabolism. Similarly, leukemias with high fatty acid oxidation capacity or enhanced autophagy can maintain mitochondrial respiration despite suppression of glutaminolysis. These observations indicate that glutamine targeting is most effective in metabolically constrained leukemias and least effective in highly plastic or therapy-adapted states. Therefore, resistance mechanisms should not be viewed as secondary caveats but as defining boundaries of glutamine addiction as a therapeutic axis.

### 6.3. Microenvironmental Buffering and Stromal Support

The bone marrow microenvironment represents a powerful extrinsic source of resistance to glutamine-directed therapies. Stromal cells, adipocytes, and immune cells within the marrow niche actively exchange metabolites with leukemic cells, including glutamine, fatty acids, and lactate [59–61]. This metabolic crosstalk can buffer leukemic cells against systemic or intracellular glutamine depletion, enabling survival despite pharmacologic inhibition of glutamine metabolism.

Hypoxia further shapes metabolic dependency within the marrow niche. Under low-oxygen conditions, leukemic cells may reduce reliance on classical oxidative glutaminolysis and instead engage reductive carboxylation or autophagy-mediated nutrient recycling [61]. Direct cell–cell interactions and cytokine signaling from stromal elements can also modulate transporter expression and metabolic enzyme activity, dynamically reshaping nutrient utilization profiles. These factors emphasize that therapeutic efficacy observed in suspension cultures may overestimate responses achievable within the complex three-dimensional marrow environment, underscoring the need for advanced preclinical models that faithfully replicate niche physiology.

### 6.4. Toxicity and Effects on Normal Hematopoiesis and Immunity

Glutamine is an essential nutrient for normal physiology, particularly for rapidly proliferating immune cells and intestinal epithelium. Systemic targeting of glutamine metabolism therefore carries an inherent risk of off-target toxicity. T cells, natural killer cells, and hematopoietic progenitors all rely on glutamine for proliferation, activation, and effector function [14–17]. Broad glutamine depletion or sustained glutaminase inhibition could compromise antileukemic immunity, impair hematopoietic recovery, or predispose patients to infection.

While early clinical studies suggest that selective GLS1 inhibition is generally tolerable, long-term effects and combination regimens targeting mitochondrial metabolism may narrow the therapeutic window [14–17]. Defining strategies that exploit differential glutamine dependency—such as intermittent dosing, metabolic cycling, or combination with agents that preferentially target leukemic mitochondria—will be critical for minimizing collateral damage to normal tissues.

### 6.5. Disease Heterogeneity and Lack of Predictive Biomarkers

A final major limitation is the heterogeneity of glutamine dependency across leukemia subtypes and among patients. Heterogeneity is evident across entities and disease states. For example, NOTCH1-driven T-ALL can exhibit enforced extracellular glutamine dependence via GS suppression [41], whereas drug-resistant ALL models have shown reduced reliance on exogenous glutamine with compensatory metabolic rewiring [46]. In AML, dependency often tracks with mitochondrial/OXPHOS-high programs enriched after therapy or in residual disease states [11–13,42,43], and can be further shaped by genotype (e.g., IDH-mutant reliance on glutamine-derived α-KG/2-HG biology) [34,36]. Clinically, these patterns suggest glutamine targeting may differ between diagnosis vs. relapse, and between proliferative vs. persistence-enriched compartments [11–13,39,40,42,43]. Not all leukemias exhibit equivalent reliance on glutamine metabolism; variability reflects differences in genetic lesions, differentiation state, metabolic wiring, and treatment history. Expression levels of IGF2BP2, GLS1, glutamine transporters, and mitochondrial activity markers vary widely among AML cases and correlate with prognosis and therapeutic response [38,58].

This heterogeneity underscores the urgent need for predictive biomarkers to guide patient selection. Integrating transcriptomic signatures, metabolic enzyme expression, and functional assessments of mitochondrial dependency may allow stratification of patients most likely to benefit from glutamine-targeted strategies. Without such biomarker-driven approaches, clinical trials risk underestimating the efficacy of glutaminedirected therapies by enrolling metabolically heterogeneous populations.

## 7. Future Directions

The growing recognition of glutamine metabolism as a contributor to leukemic growth, survival, and therapeutic resistance necessitates a shift from generalized metabolic targeting toward precision-based translational strategies. Rather than representing a universal vulnerability, glutamine addiction defines a therapeutically exploitable state in specific genetic, metabolic, and microenvironmental contexts. Its clinical utility will therefore depend on identifying leukemias that are metabolically constrained rather than highly plastic [11–13,46].

Future translational efforts must prioritize biomarker-driven stratification to identify glutamine-dependent disease states. Expression and activity of glutamine transporters and enzymes, including SLC1A5, GLS1, IGF2BP2, and glutamine synthetase, as well as functional markers of mitochondrial reliance, represent candidate parameters for patient selection [38,41,49–51]. At present, however, no validated clinical assays or metabolic thresholds exist to guide such stratification, highlighting a key barrier to implementation.

Nonetheless, several clinically feasible approaches could support early stratification efforts. Plasma amino acid profiling and LC–MS–based metabolomics have already demonstrated altered systemic glutamine levels and diagnostic/prognostic associations in acute leukemia cohorts, providing a practical foundation for incorporating glutamine-related endpoints into correlative studies [5–7]. In parallel, ongoing and recent clinical investigations of glutaminase inhibition in myeloid neoplasms have included correlative metabolic analyses, indicating that trial-integrated pharmacodynamic monitoring of glutamine pathway engagement is achievable even before formal biomarker standardization [49–51]. In this context, biomarker-driven stratification is rational as a trial design strategy, but it remains investigational and should be framed as hypothesis-testing rather than clinically established.

Equally important is the role of the bone marrow microenvironment in buffering glutamine deprivation. Stromal cells, adipocytes, and immune populations can supply alternative nutrients and promote metabolic adaptation, particularly under therapeutic pressure [59–61]. These interactions likely contribute to the discrepancy between robust preclinical efficacy and limited durability of clinical responses observed with glutamine-targeted interventions.

Given the extensive metabolic plasticity of leukemic cells, durable therapeutic benefit is unlikely to be achieved through single-agent glutamine targeting. Rational combination strategies that concurrently constrain compensatory pathways—such as fatty acid oxidation, one-carbon metabolism, or alternative antioxidant systems—may be required to exceed the adaptive capacity of leukemic cells while preserving normal hematopoiesis and immune function [11–17,59–62].

Taken together, glutamine metabolism should be viewed not as a standalone therapeutic axis but as a modulator of mitochondrial fitness, redox balance, and epigenetic state that can be leveraged to enhance the efficacy of targeted, epigenetic, or differentiation-based therapies in carefully selected leukemia subsets.

**Figure 3.**
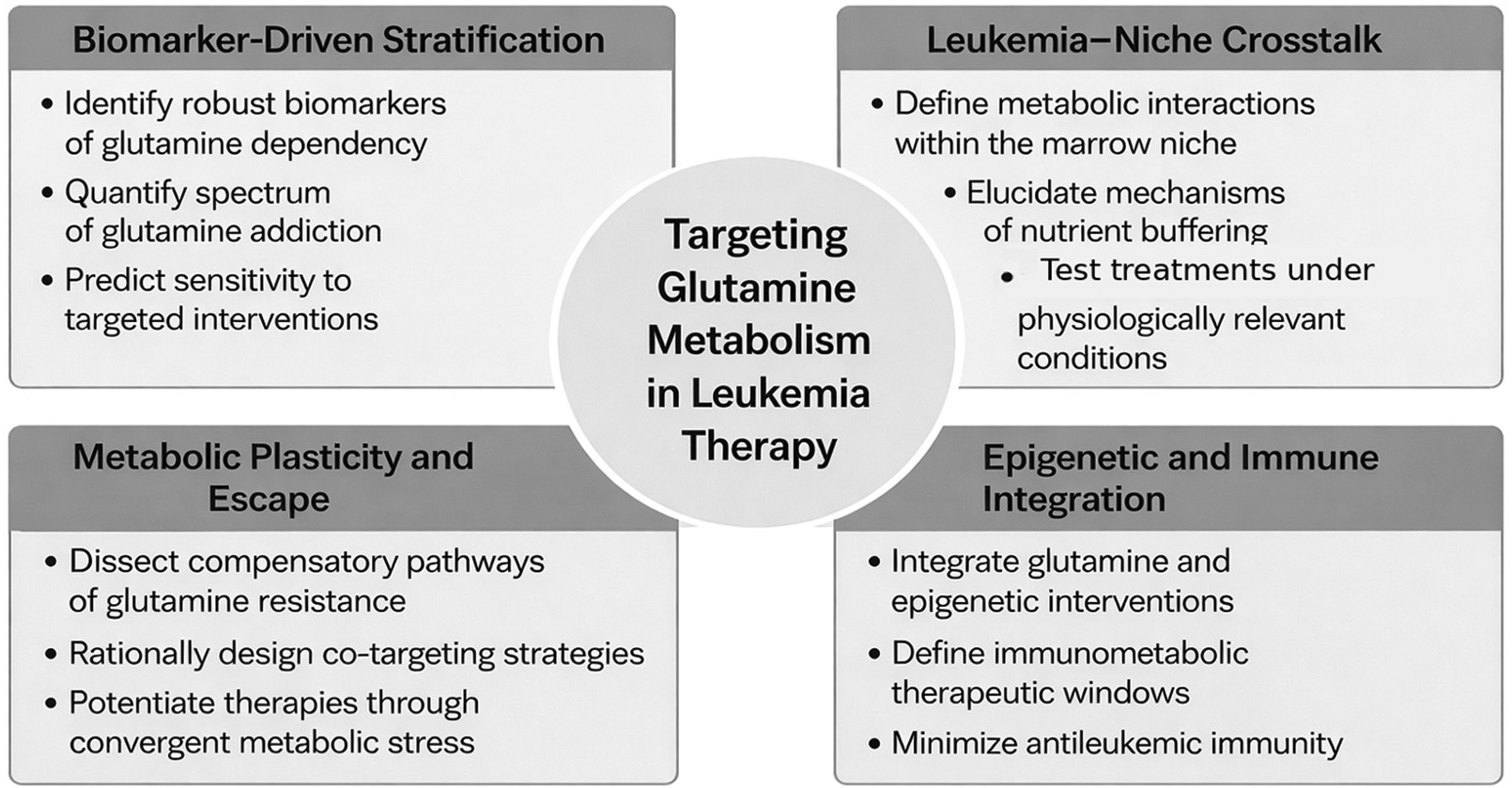
This schematic highlight key translational priorities for advancing glutamine-targeted therapies in leukemia, including biomarker-driven patient stratification, characterization of leukemia–bone marrow niche metabolic crosstalk, understanding metabolic plasticity and resistance mechanisms, and integrating glutamine metabolism with epigenetic and immune-based therapeutic strategies.

Collectively, these priorities outline a forward-looking roadmap for integrating glutamine metabolism into precision oncology for leukemia, as summarized schematically in Figure 3.

## 8. Conclusions

Glutamine metabolism has moved from a peripheral consideration to a central focus in leukemia biology. It supports energy production, biosynthesis, redox balance, epigenetic regulation and stemness, and its disruption can profoundly impair leukemic cell survival. Multiple layers of regulation, including oncogenic transcription factors, m^6A-dependent RNA binding proteins and microenvironmental cues, converge to enforce glutamine addiction in specific leukemia subtypes. Preclinical data provide compelling evidence that glutaminase inhibition and related strategies can produce significant antileukemic effects, especially in combination with agents that exploit the resulting redox crisis or target complementary pathways.

At the same time, the very ubiquity of glutamine as a nutrient, the plasticity of cancer metabolism and the support provided by the bone marrow niche create formidable barriers to therapeutic exploitation. Overcoming these challenges will require biomarker-driven patient selection, sophisticated preclinical models that recapitulate the marrow microenvironment and carefully designed combination regimens that balance efficacy against toxicity.

Rather than representing a universal vulnerability, glutamine addiction defines a therapeutically exploitable state in specific genetic, metabolic, and microenvironmental contexts. Its clinical utility will depend on identifying leukemias that are metabolically constrained rather than highly plastic.

Concluding remarks: (i) Glutamine metabolism supports leukemia survival through integrated TCA anaplerosis/OXPHOS, redox buffering, and epigenetic control [8,11–13,35,36]. (ii) “Glutamine addiction” is a conditional state that emerges in defined genetic and microenvironmental contexts and is frequently attenuated by metabolic plasticity [11–13,46,63]. (iii) Clinical translation will require state-aware combinations and trial-embedded metabolic biomarkers rather than single-agent glutamine targeting [11–13,49–51,59–61].

## Figures and Tables

**Table 1.** Main glutamine transporters and their functional interrelationships in leukemia.

Transporter/Family	Common Name	Directionality	Key Substrate(s)	Role in Leukemia	Functional Coupling	Key References
SLC1A5	ASCT2	Influx (Na^+^-dependent)	Glutamine	Primary glutamine entry; supports mitochondrial fueling and survival	Supplies intracellular glutamine for LAT1 exchange and mTORC1 activation	[10,22]
SLC38 family	SNATs	Influx/Efflux (context-dependent)	Glutamine, neutral AAs	Maintains cytosolic glutamine under nutrient stress	Compensates for SLC1A5; feeds exchange systems	[22]
SLC7A5 + SLC3A2	LAT1 (CD98)	Obligate exchanger	Leucine ↔ Glutamine	Enables anabolic signaling	Requires intracellular glutamine to drive leucine uptake → mTORC1	[23,24]

Abbreviations: ASCT2, alanine–serine–cysteine transporter 2; LAT1, L-type amino acid transporter 1; mTORC1, mechanistic target of rapamycin complex 1; SNAT, sodium-coupled neutral amino acid transporter.

**Table 2.** **C**ore Glutamine Metabolic Pathways and Functional Outputs in Leukemia.

Metabolic Step	Key Enzyme/Transporter	Compartment	Major Product(s)	Primary Functional Output	Leukemia Context	Key References
Glutamine uptake	SLC1A5 (ASCT2)	Plasma membrane	Intracellular glutamine	Substrate supply for metabolism	AML, ALL	[10,19–21]
Homeostatic transport	SLC38 (SNATs)	Plasma membrane	Glutamine flux balance	Nutrient buffering	Bone marrow niche	[22]
Amino acid exchange	LAT1 (SLC7A5)	Plasma membrane	Leucine ↔ Glutamine	mTORC1 activation	Proliferative AML	[23,24]
Glutaminolysis	GLS1	Mitochondria	Glutamate	TCA entry, redox support	OXPHOS-high AML	[8,26,31]
Anaplerosis	GLDH / aminotransferases	Mitochondria	α-ketoglutarate	Sustains TCA and OXPHOS	Chemoresistant AML	[11–13,26]
Redox buffering	GSH synthesis enzymes	Cytosol/mitochondria	Glutathione	ROS detoxification	LSCs, resistance	[8,31–33]
Biosynthesis	Purine/pyrimidine enzymes	Cytosol	Nucleotides	Supports proliferation	Cycling blasts	[26,28]
Epigenetic regulation	TET / JmjC enzymes	Nucleus	Demethylated chromatin	Controls differentiation	IDH-mut AML	[34–36]

Abbreviations: GSH, glutathione; GLS, glutaminase; LSC, leukemic stem cell; OXPHOS, oxidative phosphorylation; ROS, reactive oxygen species; TCA, tricarboxylic acid cycle.

**Table 3.** Key Roles of Glutamine in Leukemia Cell Biology.

Functional Domain	Role of Glutamine	Key Pathway/Node	Biological Consequence	Resistance/Limitations	Key References
Bioenergetics	Anaplerosis into TCA	GLS ↔ α-KG	Supports OXPHOS and ATP production	FAO compensation; autophagy	[11–13,26]
Biosynthesis	Nucleotide synthesis	Purine/pyrimidine pathways	Supports proliferation	Serine-glycine/1C metabolism	[26,28,37]
Redox	GSH synthesis	Glutamate → GSH	ROS buffering	Alternative antioxidant systems	[8,31–33]
Epigenetics	α-KG cofactor	TET/JmjC enzymes	Controls differentiation	IDH mutations, 2-HG	[34–36]
Stemness	Mitochondrial support	OXPHOS-high LSCs	Self-renewal, MRD	Metabolic plasticity	[38–40]

Abbreviations: α-KG, α-ketoglutarate; LSC, leukemic stem cell; TCA, tricarboxylic acid cycle.

**Table 4.** Molecular Regulators of Glutamine Metabolism in Leukemia.

Regulatory Layer	Regulator	Mechanism	Leukemia Context	Failure Context	Key References
Oncogenic TF	MYC	Induces SLC1A5, GLS	AML, MYC-high	MYC-low leukemias	[34,44]
Nutrient sensing	mTORC1	Leucine exchange via LAT1	Proliferative AML	Nutrient-poor niche	[23,24]
RNA regulation	IGF2BP2	m6A-dependent stabilization	AML stem-like cells	IGF2BP2-low AML	[38]
Synthesis loss	GS suppression	Enforced addiction	NOTCH1 TALL	GS-high leukemias	[41]
Therapy stress	FLT3i	Shift to OXPHOS/glutamine	FLT3-mut AML	FAO-adapted AML	[42,43]

Abbreviations: AML, acute myeloid leukemia; FAO, fatty acid oxidation; FLT3i, FLT3 inhibitor; GS, glutamine synthetase; IGF2BP2, insulin-like growth factor 2 mRNA-binding protein 2; mTORC1, mechanistic target of rapamycin complex 1; OXPHOS, oxidative phosphorylation; T-ALL, T-cell acute lymphoblastic leukemia.

**Table 5.** Therapeutic Strategies Targeting Glutamine in Leukemia.

Strategy	Target	Example Agents	Main Effect	Resistance Mechanisms	Clinical Status	Key References
GLS inhibition	GLS1	Telaglenastat (CB-839)	↓ TCA, ↓ GSH	ASNS activity, FAO	Phase I-II	[8,31,49–51]
Transport block	SLC1A5	V-9302	↓ uptake	Transport redundancy	Preclinical	[10,52]
Enzymatic depletion	Asparaginase	Peg-ASNase	↓ Asn + Gln	Toxicity, immunity	Standard in ALL	[53]
Combinations	Mitochondria/redox	Venetoclax, ATO	Synergy	Plasticity	Early trials	[54,55]

Abbreviations: ALL, acute lymphoblastic leukemia; ASNS, asparagine synthetase; FAO, fatty acid oxidation; GLS1, glutaminase 1; GSH, glutathione; SLC1A5, solute carrier family 1 member 5; TCA, tricarboxylic acid cycle.

## Data Availability

No new data were created or analyzed in this study. Data sharing is not applicable to this article.

## References

[1] Yang L; Venneti S; Nagrath D Glutaminolysis: A hallmark of cancer metabolism. Annu. Rev. Biomed. Eng 2017, 19, 163–194. 28301735 10.1146/annurev-bioeng-071516-044546

[2] Schulze A; Harris AL How cancer metabolism is tuned for proliferation and vulnerable to disruption. Nature 2012, 491, 364–373. 23151579 10.1038/nature11706

[3] Panuzzo C; Jovanovski A; Pergolizzi B; Pironi L; Stanga S; Fava C; Cilloni D Mitochondria: A galaxy in the hematopoietic and leukemic stem cell universe. Int. J. Mol. Sci 2020, 21, 3928. 32486249 10.3390/ijms21113928 PMC7312164

[4] Basak NP; Banerjee S Mitochondrial dependency in progression of acute myeloid leukemia. Mitochondrion 2015, 21, 41–48. 25640960 10.1016/j.mito.2015.01.006

[5] Wang Y; Zhang L; Chen W-L; Wang J-H; Li N; Li J-M; Mi J-Q; Zhang W-N; Li Y; Wu S-F; Rapid diagnosis and prognosis of de novo acute myeloid leukemia by serum metabonomic analysis. J. Proteome Res 2013, 12, 4393–4401. 23998518 10.1021/pr400403p

[6] Rudman D; Vogler WR; Howard CH; Gerron GG Observations on the plasma amino acids of patients with acute leukemia. Cancer Res 1971, 31, 1159–1165. 5285976

[7] Wang D; Tan G; Wang H; Chen P; Hao J; Wang Y Identification of novel serum biomarker for the detection of acute myeloid leukemia based on liquid chromatography-mass spectrometry. J. Pharm. Biomed. Anal 2019, 166, 357–363. 30690249 10.1016/j.jpba.2019.01.022

[8] Gregory MA; Nemkov T; Park HJ; Zaberezhnyy V; Gehrke S; Adane B; Jordan CT; Hansen KC; D Alessandro A; DeGregori J Targeting glutamine metabolism and redox state for leukemia therapy. Clin. Cancer Res. Off. J. Am. Assoc. Cancer Res 2019, 25, 4079–4090. 10.1158/1078-0432.CCR-18-3223 PMC6642698 30940653

[9] Döhner H; Weisdorf DJ; Bloomfield CD Acute myeloid leukemia. N. Engl. J. Med 2015, 373, 1136–1152. 26376137 10.1056/NEJMra1406184

[10] Willems L; Jacque N; Jacquel A; Neveux N; Maciel TT; Lambert M; Schmitt A; Poulain L; Green AS; Uzunov M; Inhibiting glutamine uptake represents an attractive new strategy for treating acute myeloid leukemia. Blood 2013, 122, 3521–3532. 24014241 10.1182/blood-2013-03-493163 PMC3829119

[11] Culp-Hill R; D Alessandro A; Pietras EM Extinguishing the embers: Targeting AML metabolism. Trends Mol. Med 2021, 27, 332–344. 33121874 10.1016/j.molmed.2020.10.001 PMC8005405

[12] Lagadinou ED; Sach A; Callahan K; Rossi RM; Neering SJ; Minhajuddin M; Ashton JM; Pei S; Grose V; O Dwyer KM; BCL-2 inhibition targets oxidative phosphorylation and selectively eradicates quiescent human leukemia stem cells. Cell Stem Cell 2013, 12, 329–341. 23333149 10.1016/j.stem.2012.12.013 PMC3595363

[13] Farge T; Saland E; de Toni F; Aroua N; Hosseini M; Perry R; Bosc C; Sugita M; Stuani L; Fraisse M; Chemotherapy-resistant human acute myeloid leukemia cells are not enriched for leukemic stem cells but require oxidative metabolism. Cancer Discov 2017, 7, 716–735. 28416471 10.1158/2159-8290.CD-16-0441 PMC5501738

[14] van Gastel N; Spinelli JB; Sharda A; Schajnovitz A; Baryawno N; Rhee C; Oki T; Grace E; Soled HJ; Milosevic J; Induction of a timed metabolic collapse to overcome cancer chemoresistance. Cell Metab 2020, 32, 391–403.e6. 32763164 10.1016/j.cmet.2020.07.009 PMC8397232

[15] Ma G; Zhang Z; Li P; Zhang Z; Zeng M; Liang Z; Li D; Wang L; Chen Y; Liang Y; Reprogramming of glutamine metabolism and its impact on immune response in the tumor microenvironment. Cell Commun. Signal. CCS 2022, 20, 114. 35897036 10.1186/s12964-022-00909-0 PMC9327201

[16] Sornsuvit C; Komindr S; Chuncharunee S; Wanikiat P; Archararit N; Santanirand P Pilot Study: Effects of parenteral glutamine dipeptide supplementation on neutrophil functions and prevention of chemotherapy-induced side-effects in acute myeloid leukaemia patients. J. Int. Med. Res 2008, 36, 1383–1391. 19094450 10.1177/147323000803600628

[17] Liu P-S; Wang H; Li X; Chao T; Teav T; Christen S; Di Conza G; Cheng W-C; Chou C-H; Vavakova M; *α*-ketoglutarate orchestrates macrophage activation through metabolic and epigenetic reprogramming. Nat. Immunol 2017, 18, 985–994. 28714978 10.1038/ni.3796

[18] Darmaun D; Matthews DE; Bier DM Glutamine and glutamate kinetics in humans. Am. J. Physiol 1986, 251, E117–E126. 2873746 10.1152/ajpendo.1986.251.1.E117

[19] Cormerais Y; Massard PA; Vucetic M; Giuliano S; Tambutté E; Durivault J; Vial V; Endou H; Wempe MF; Parks SK; The glutamine transporter ASCT2 (SLC1A5) promotes tumor growth independently of the amino acid transporter LAT1 (SLC7A5). J. Biol. Chem 2018, 293, 2877–2887. 29326164 10.1074/jbc.RA117.001342 PMC5827425

[20] Ni F; Yu W-M; Li Z; Graham DK; Jin L; Kang S; Rossi MR; Li S; Broxmeyer HE; Qu C-K Critical role of ASCT2-mediated amino acid metabolism in promoting leukaemia development and progression. Nat. Metab 2019, 1, 390–403. 31535081 10.1038/s42255-019-0039-6 PMC6750232

[21] Schulte ML; Fu A; Zhao P; Li J; Geng L; Smith ST; Kondo J; Coffey RJ; Johnson MO; Rathmell JC; Pharmacological blockade of ASCT2-dependent glutamine transport leads to anti-tumor efficacy in preclinical models. Nat. Med 2018, 24, 194–202. 29334372 10.1038/nm.4464 PMC5803339

[22] Polet F; Martherus R; Corbet C; Pinto A; Feron O Inhibition of glucose metabolism prevents glycosylation of the glutamine transporter ASCT2 and promotes compensatory LAT1 upregulation in leukemia cells. Oncotarget 2016, 7, 46371–46383. 27344174 10.18632/oncotarget.10131 PMC5216804

[23] Durán RV; Oppliger W; Robitaille AM; Heiserich L; Skendaj R; Gottlieb E; Hall MN Glutaminolysis activates RagmTORC1 signaling. Mol. Cell 2012, 47, 349–358. 22749528 10.1016/j.molcel.2012.05.043

[24] Wang L; Zhu L; Wu K; Chen Y; Lee D-Y; Gucek M; Sack MN Mitochondrial general control of amino acid synthesis 5 Like 1 regulates glutaminolysis, mTORC1 activity and murine liver regeneration. Hepatology 2020, 71, 643–657. 31344750 10.1002/hep.30876 PMC7465484

[25] Dernie F Characterisation of a mitochondrial glutamine transporter provides a new opportunity for targeting glutamine metabolism in acute myeloid leukaemia. Blood Cells Mol. Dis 2021, 88, 102422. 32197941 10.1016/j.bcmd.2020.102422

[26] Rex MR; Williams R; Birsoy K; Ta Llman MS; Stahl M Targeting mitochondrial metabolism in acute myeloid leukemia. Leuk. Lymphoma 2022, 63, 530–537. 34704521 10.1080/10428194.2021.1992759

[27] Gao M; Yi J; Zhu J; Minikes AM; Monian P; Thompson CB; Jiang X Role of mitochondria in ferroptosis. Mol. Cell 2019, 73, 354–363.e3. 30581146 10.1016/j.molcel.2018.10.042 PMC6338496

[28] Zuo F; Yu J; He X Single-cell metabolomics in hematopoiesis and hematological malignancies. Front. Oncol 2022, 12, 931393. 35912231 10.3389/fonc.2022.931393 PMC9326066

[29] DeBerardinis RJ; Mancuso A; Daikhin E; Nissim I; Yudkoff M; Wehrli S; Thompson CB Beyond aerobic glycolysis: Transformed cells can engage in glutamine metabolism that exceeds the requirement for protein and nucleotide synthesis. Proc. Natl. Acad. Sci. USA 2007, 104, 19345–19350. 18032601 10.1073/pnas.0709747104 PMC2148292

[30] Sharma ND; Keewan E; Matlawska-Wasowska K Metabolic reprogramming and cell adhesion in acute leukemia adaptation to the CNS niche. Front. Cell Dev. Biol 2021, 9, 767510. 34957100 10.3389/fcell.2021.767510 PMC8703109

[31] Jacque N; Ronchetti AM; Larrue C; Meunier G; Birsen R; Willems L; Saland E; Decroocq J; Maciel TT; Lambert M; Targeting glutaminolysis has antileukemic activity in acute myeloid leukemia and synergizes with BCL-2 inhibition. Blood 2015, 126, 1346–1356. 26186940 10.1182/blood-2015-01-621870 PMC4608389

[32] Romo-González M; Ijurko C; Hernández-Hernández Á Reactive oxygen species and metabolism in leukemia: A dangerous liaison. Front. Immunol 2022, 13, 889875. 35757686 10.3389/fimmu.2022.889875 PMC9218220

[33] Chen Y-F; Liu H; Luo X-J; Zhao Z; Zou Z-Y; Li J; Lin X-J; Liang Y The roles of reactive oxygen species (ROS) and autophagy in the survival and death of leukemia cells. Crit. Rev. Oncol. Hematol 2017, 112, 21–30. 28325262 10.1016/j.critrevonc.2017.02.004

[34] Emadi A; Jun SA; Tsukamoto T; Fathi AT; Minden MD; Dang CV Inhibition of glutaminase selectively suppresses the growth of primary acute myeloid leukemia cells with IDH mutations. Exp. Hematol 2014, 42, 247–251. 24333121 10.1016/j.exphem.2013.12.001

[35] Sánchez-Mendoza SE; Rego EM Targeting the mitochondria in acute myeloid leukemia. Appl. Cancer Res 2017, 37, 22.

[36] Ward PS; Patel J; Wise DR; Abdel-Wahab O; Bennett BD; Coller HA; Cross JR; Fantin VR; Hedvat CV; Perl AE; The common feature of leukemia-associated IDH1 and IDH2 mutations is a neomorphic enzymatic activity that converts α-ketoglutarate to 2-hydroxyglutarate. Cancer Cell 2010, 17, 225–234. 20171147 10.1016/j.ccr.2010.01.020 PMC2849316

[37] Polet F; Corbet C; Pinto A; Rubio LI; Martherus R; Bol V; Drozak X; Grégoire V; Riant O; Feron O Reducing the serine availability complements the inhibition of the glutamine metabolism to block leukemia cell growth. Oncotarget 2016, 7, 1765–1776. 26625201 10.18632/oncotarget.6426 PMC4811496

[38] Weng H; Huang F; Yu Z; Chen Z; Prince E; Kang Y; Zhou K; Li W; Hu J; Fu C; The m6A reader IGF2BP2 regulates glutamine metabolism and represents a therapeutic target in acute myeloid leukemia. Cancer Cell 2022, 40, 1566–1582.e10. 36306790 10.1016/j.ccell.2022.10.004 PMC9772162

[39] Srinivasan Rajsri K; Roy N; Chakraborty S Acute Myeloid Leukemia Stem Cells in Minimal/Measurable Residual Disease Detection. Cancers 2023, 15, 2866. Erratum in Cancers 2024, 16, 954. 10.3390/cancers15102866. 37345204 PMC10216329

[40] Álvarez N; Martín A; Dorado S; Colmenares R; Rufián L; Rodríguez M; Giménez A; Carneros L; Sanchez R; Carreño G; Detection of minimal residual disease in acute myeloid leukemia: Evaluating utility and challenges. Front. Immunol 2024, 15, 1252258. 38938565 10.3389/fimmu.2024.1252258 PMC11210172

[41] Nguyen TL; Nokin M-J; Terés S; Tomé M; Bodineau C; Galmar O; Pasquet J-M; Rousseau B; van Liempd S; Falcon-Perez JM; Downregulation of glutamine synthetase, not glutaminolysis, is responsible for glutamine addiction in Notch1-driven acute lymphoblastic leukemia. Mol. Oncol 2021, 15, 1412–1431. 33314742 10.1002/1878-0261.12877 PMC8096784

[42] Firmanty P; Chomczyk M; Dash S; Konopleva M; Baran N Feasibility and Safety of Targeting Mitochondria Function and Metabolism in Acute Myeloid Leukemia. Curr. Pharmacol. Rep 2024, 10, 388–404. 40756330 10.1007/s40495-024-00378-8 PMC12314886

[43] Baran N; Lodi A; Dhungana Y; Herbrich S; Collins M; Sweeney S; Pandey R; Skwarska A; Patel S; Tremblay M; Inhibition of mitochondrial complex I reverses NOTCH1-driven metabolic reprogramming in T-cell acute lymphoblastic leukemia. Nat. Commun 2022, 13, 2801. 35589701 10.1038/s41467-022-30396-3 PMC9120040

[44] Gao P; Tchernyshyov I; Chang T-C; Lee Y-S; Kita K; Ochi T; Zeller K; De Marzo AM; Van Eyk JE; Mendell JT; c-Myc suppression of miR-23 enhances mitochondrial glutaminase and glutamine metabolism. Nature 2009, 458, 762–765. 19219026 10.1038/nature07823 PMC2729443

[45] Mao H; Wen Y; Yu Y; Li H; Wang J; Sun B Bioinspired nanocatalytic tumor therapy by simultaneous reactive oxygen species generation enhancement and glutamine pathway-mediated glutathione depletion. J. Mater. Chem. B 2022, 11, 131–143. 36484247 10.1039/d2tb02194c

[46] Stäubert C; Bhuiyan H; Lindahl A; Broom OJ; Zhu Y; Islam S; Linnarsson S; Lehtiö J; Nordström A Rewired metabolism in drug-resistant leukemia cells: A metabolic switch hallmarked by reduced dependence on exogenous glutamine. J. Biol. Chem 2015, 290, 8348–8359. 25697355 10.1074/jbc.M114.618769 PMC4375488

[47] Mannan A; Germon ZP; Chamberlain J; Sillar JR; Nixon B; Dun MD Reactive oxygen species in acute lymphoblastic leukaemia: Reducing radicals to refine responses. Antioxidants 2021, 10, 1616. 34679751 10.3390/antiox10101616 PMC8533157

[48] Birben E; Sahiner UM; Sackesen C; Erzurum S; Kalayci O Oxidative stress and antioxidant defense. World Allergy Organ. J 2012, 5, 9–19. 23268465 10.1097/WOX.0b013e3182439613 PMC3488923

[49] Konopleva M; DiNardo C; Bhagat T; Baran N; Lodi A; Saxena K; Cai T; Su X; Skwarska A; Guerra V; Glutaminase inhibition in combination with azacytidine in myelodysplastic syndromes: Clinical efficacy and correlative analyses. Res. Sq 2023, rs.3.rs-2518774. 10.21203/rs.3.rs-2518774/v1. PMC13318259 39300320

[50] Usart M; Hansen N; Stetka J; Almeida Fonseca T; Guy A; Kimmerlin Q; Rai S; Hao-Shen H; Roux J; Dirnhofer S; The glutaminase inhibitor CB-839 targets metabolic dependencies of JAK2-mutant hematopoiesis in MPN. Blood Adv 2024, 8, 2312–2325. 38295283 10.1182/bloodadvances.2023010950 PMC11117009

[51] Timofeeva N; Ayres ML; Baran N; Santiago-O Farrill JM; Bildik G; Lu Z; Konopleva M; Gandhi V Preclinical investigations of the efficacy of the glutaminase inhibitor CB-839 alone and in combinations in chronic lymphocytic leukemia. Front. Oncol 2023, 13, 1161254. 37228498 10.3389/fonc.2023.1161254 PMC10203524

[52] Jin J; Byun JK; Choi YK; Park KG Targeting glutamine metabolism as a therapeutic strategy for cancer. Exp. Mol. Med 2023, 55, 706–715. 37009798 10.1038/s12276-023-00971-9 PMC10167356

[53] Wang Z; Liu M; Yang Q Glutamine and leukemia research: Progress and clinical prospects. Discov. Oncol 2024, 15, 391. 39215845 10.1007/s12672-024-01245-0 PMC11365919

[54] Gregory MA; D Alessandro A; Alvarez-Calderon F; Kim J; Nemkov T; Adane B; Rozhok AI; Kumar A; Kumar V; Pollyea DA; ATM/G6PD-driven redox metabolism promotes FLT3 inhibitor resistance in acute myeloid leukemia. Proc. Natl. Acad. Sci. USA 2016, 113, E6669–E6678. 27791036 10.1073/pnas.1603876113 PMC5086999

[55] Wang Y; Huang B; Liang T; Jiang L; Wu M; Liu X; Zhu M; Song X; Zhao N; Wei H; Venetoclax acts as an immunometabolic modulator to potentiate adoptive NK cell immunotherapy against leukemia. Cell Rep. Med 2024, 5, 101580. 38776913 10.1016/j.xcrm.2024.101580 PMC11228450

[56] Dembitz V; Gallipoli P The Role of Metabolism in the Development of Personalized Therapies in Acute Myeloid Leukemia. Front. Oncol 2021, 11, 665291. 34094959 10.3389/fonc.2021.665291 PMC8170311

[57] Fan Y; Xue H; Li Z; Huo M; Gao H; Guan X Exploiting the Achilles heel of cancer: Disrupting glutamine metabolism for effective cancer treatment. Front. Pharmacol 2024, 15, 1345522. 38510646 10.3389/fphar.2024.1345522 PMC10952006

[58] Corti A; Dominici S; Piaggi S; Belcastro E; Chiu M; Taurino G; Pacini S; Bussolati O; Pompella A γ-Glutamyltransferase enzyme activity of cancer cells modulates L-γ-glutamyl-p-nitroanilide (GPNA) cytotoxicity. Sci. Rep 2019, 9, 891. 30696905 10.1038/s41598-018-37385-x PMC6351548

[59] Tabe Y; Konopleva M; Andreeff M Fatty acid metabolism, bone marrow adipocytes, and AML. Front. Oncol 2020, 10, 155. 32133293 10.3389/fonc.2020.00155 PMC7040225

[60] Röhrig F; Schulze A The multifaceted roles of fatty acid synthesis in cancer. Nat. Rev. Cancer 2016, 16, 732–749. 27658529 10.1038/nrc.2016.89

[61] Beloribi-Djefaflia S; Vasseur S; Guillaumond F Lipid metabolic reprogramming in cancer cells. Oncogenesis 2016, 5, e189. 26807644 10.1038/oncsis.2015.49 PMC4728678

[62] Chen Q; Kirk K; Shurubor YI; Zhao D; Arreguin AJ; Shahi I; Valsecchi F; Primiano G; Calder EL; Carelli V; Rewiring of glutamine metabolism is a bioenergetic adaptation of human cells with mitochondrial DNA mutations. Cell Metab 2018, 27, 1007–1025.e5. 29657030 10.1016/j.cmet.2018.03.002 PMC5932217

[63] Tabe Y; Lorenzi PL; Konopleva M Amino acid metabolism in hematologic malignancies and the era of targeted therapy. Blood 2019, 134, 1014–1023. 31416801 10.1182/blood.2019001034 PMC6764269

